# Long-Term Postoperative Outcomes of Sinus Venosus Defect

**DOI:** 10.3390/jcm15145472

**Published:** 2026-07-13

**Authors:** Mariama Touray, Magalie Ladouceur, Judith Bouchardy, Markus Schwerzmann, Matthias Greutmann, Daniel Tobler, Reto Engel, Harald Gabriel, Caroline Blanche, Etienne Pruvot, Nicole Sekarski, Tobias Rutz

**Affiliations:** 1 Service of Cardiology, Heart and Vessel Department, Lausanne University Hospital, University of Lausanne, 1005 Lausanne, Switzerland; judith.bouchardy@chuv.ch (J.B.); etienne.pruvot@chuv.ch (E.P.); tobias.rutz@chuv.ch (T.R.); 2 Adult Congenital Heart Disease Unit, Centre de Référence des Malformations Cardiaques Congénitales Complexes, M3C, Department of Cardiology, Hôpital Européen Georges Pompidou, Hôpitaux de Paris, 75015 Paris, France; magalie.ladouceur@gmail.com; 3 Department of Congenital Heart Disease, M3C Network for Complex Congenital Heart Diseases, Marie Lannelongue Hospital, Paris-Saclay University, 92350 Le Plessis-Robinson, France; 4 INSERM UMR_S 999 Pulmonary Hypertension: Pathophysiology and Novel Therapies (HPPIT), Paris Saclay University, 92350 Le Plessis-Robinson, France; 5 Cardiology Unit, University Hospitals of Geneva, 1211 Geneva, Switzerland; carolineblanche@ccnyon.ch; 6 Department of Cardiology, Center for Congenital Heart Disease, Inselspital, University of Bern, 3010 Bern, Switzerland; mschwerzmann@ottawaheart.ca; 7 Department of Cardiology, University Heart Center, University of Zurich, 8006 Zürich, Switzerland; matthias.greutmann@hirslanden.ch; 8 Department of Cardiology, University Hospital of Basel, University of Basel, 4001 Basel, Switzerland; daniel.tobler@usb.ch; 9 Department of Cardiology, Kantonsspital St. Gallen, 9007 St. Gallen, Switzerland; reto.engel@h-och.ch; 10 Department of Cardiology, Vienna General Hospital, Medical University of Vienna, 1090 Vienna, Austria; gabriel.harald@gmx.at; 11 Paediatric Cardiology Unit, Women-Mother-Child Department, Lausanne University Hospital, University of Lausanne, 1005 Lausanne, Switzerland; nicole.sekarski@chuv.ch

**Keywords:** sinus venosus defect, atrial septal defects, long-term outcomes

## Abstract

**Background/Objectives**: Despite advances in transcatheter therapy, surgery remains the gold standard treatment for sinus venosus defect (SVD). However, data on long-term outcomes following surgical repair remain scarce. This multicenter study evaluates the long-term outcomes of surgically treated SVD patients with comparison to surgically and percutaneously treated secundum atrial septal defect (ASD). **Methods**: Clinical, surgical, imaging and invasive data were retrospectively reviewed from eight centers in Europe. **Results**: A total of 209 patients were included, of whom 80 were surgically treated SVD, 57 surgically treated secundum ASD and 72 percutaneously treated secundum ASD. Operation for SVD mainly occurred in adulthood, with a median age of 28.5 years (2–68 years). During follow-up, the observed reoperation rate was higher in the SVD cohort than ASD cohorts (*p* = 0.033), despite shorter follow-up period in the SVD cohort. Reoperations, in the SVD cohort, occurred at a median of 28 years (1–50 years) after index surgery. The need for pacemaker implantation was similar between groups (*p* = 0.301). The prevalence of late atrial fibrillation did not differ between groups (*p* = 0.588). Surgically treated SVD tended to have a higher prevalence of atrial flutter and atrial tachycardia, with a significantly higher rate of electrophysiological studies than percutaneously treated secundum ASD (*p* = 0.013), similar to surgically treated secundum ASD (*p* = 0.405). **Conclusions**: Long-term follow-up of surgically treated SVD is essential to monitor for reintervention and arrhythmic complications.

## 1. Introduction

Interatrial defects represent the most common congenital heart disease (CHD) diagnosed in adults with a prevalence of approximately 1.4 per 1000 individuals [1,2]. Sinus venosus defect (SVD), first described in 1858, accounts for approximately 5% of interatrial defects, with more than 90% classified as the superior type [2]. Superior SVD is characterized by communication between the right and left atrium superiorly and posteriorly to the fossa ovalis, at the superior cavo-atrial junction [3]. The inferior SVD is defined by communication in the inferior part of the interatrial septum and the inferior vena cava. SVD fundamentally differentiates from the other types of interatrial defects as the atrial septum primum and secundum are usually preserved [3]. Moreover, SVD is associated with partial anomalous pulmonary venous connection (PAPVC) in 88% [2,4].

As for other types of interatrial defects, SVDs cause a left-to-right shunt leading to right heart dilatation and increased pulmonary blood flow. Long-term risks if left untreated include progressive right ventricular (RV) dilatation and eventually failure, progressive tricuspid regurgitation, pulmonary arterial hypertension and arrhythmias [5].

Surgery remains the gold standard treatment for patients with SVD associated with PAPVC5. Due to the heterogeneous anatomical variations, multiple surgical techniques have been developed, reflecting the inherent complexity of this defect. Studies have shown the safety of these surgical techniques [4,6]. However, data on the long-term outcomes are limited. As transcatheter therapies evolve, reliable outcome data is crucial to guide and contextualize future prospective research. The present study hypothesizes that outcomes for surgically treated SVD may differ from those of other interatrial defects closures. This could be due to the greater anatomical complexity of SVD, the frequent association with defects such as PAPVC that can be overlooked on transthoracic echocardiography, and the tendency for SVD patients to develop pulmonary hypertension at an earlier stage compared to those with secundum atrial septal defect (ASD) [7]. A unique feature of the present study is, indeed, its comparative approach: rather than solely reporting the long-term outcomes of patients with surgically treated SVD, we also directly compare these outcomes with those observed in other types of interatrial defects [6]. Thus, we aim to explore the long-term outcomes of adults with repaired SVDs compared to adults with surgically and percutaneously repaired secundum ASD.

## 2. Materials and Methods

Adult patients diagnosed with SVD associated with PAPVC included in the Swiss Adult Congenital Heart Registry (SACHER) database in six centers in Switzerland—as well as in Vienna, Austria, and one further center in Paris, France—were included in the study. The study methods have been published for a study on patients with total anomalous pulmonary venous connection [8]. All SVDs were associated with PAPVC. Patients with associated complex CHD lesions (e.g., transposition of the great arteries, tetralogy of Fallot) were excluded. Adult patients with isolated secundum ASD were included for comparison of SVD patients.

Medical reports, ECG and Holter-ECG, surgical notes, reports from cardiac magnetic resonance (CMR), cardiac catheterization, echocardiography, exercise testing and cardio-pulmonary exercise testing (CPET) were collected retrospectively for each patient for the pre- and perioperative periods, and the last follow-up when available.

The study was conducted according to the guidelines of the Declaration of Helsinki, and approved by the Ethics Committee Canton of Vaud, Switzerland, number 2018-00285 on 15 August 2018.

### 2.1. Hemodynamic Parameters

Hemodynamic parameters were extracted from reports of echocardiography, CMR or right heart catheterization (RHC). The degree of shunt was defined according to the pulmonary–systemic (Qp:Qs) blood flow ratio evaluated on CMR and/or RHC. Pulmonary hypertension was defined, according to the ESC 2022 guidelines, by a systolic pulmonary artery pressure (PAP) > 35 mmHg and tricuspid regurgitation velocity > 2.8 m/sec by TTE or as determined by a mean PAP of >20 mmHg on RHC [9]. The left and right ventricular (RV) dimensions and volumes were obtained from echocardiography and CMR reports, respectively, and considered dilated according to the published reference values [10,11]. TTE RV systolic function was evaluated by tricuspid annular plane systolic excursion (TAPSE, mm), pulsed Doppler peak S’ (cm/s) and visual assessment. TAPSE was considered abnormal under 16 mm and pulsed Doppler peak S’ below 10 m/s [12]. RV dilatation was defined by a basal diameter > 41 mm, or a telediastolic surface of >11.5 cm^2^/m^2^ for women and >12.6 cm^2^/m^2^ for men [12].

Prevalence of arrhythmias was assessed through resting ECG, Holter-ECG report, CPET and electrophysiological study (EPS). Only sustained supraventricular arrhythmias—defined as episodes lasting longer than 30 s—were included in the analysis. Supraventricular arrhythmias included atrial fibrillation, atrial flutter and atrial tachycardia. Late post-operative arrhythmias were defined as arrhythmias occurring more than one month after the surgery. Treatment of arrhythmias were recorded: medication, ablation during EPS or with a pacemaker.

### 2.2. Surgical Data

Three different techniques were reported as follows:

1. The single-patch technique: one patch was used to close the SVD and redirect the PAPVC to the left atrium [13].
2. The double-patch technique: a patch is used to widen the supervisor vena cava and one to close the defect and redirect the anomalous pulmonary veins [13].
3. The Warden technique: consists of dividing the superior vena cava into two and redirecting the flow of the anomalous pulmonary veins with a patch though the exiting interatrial communication [14].

### 2.3. Outcomes

The outcomes assessed in this study were exploratory based on available literature. The primary exploratory outcome was the need for reoperation and the prevalence of supraventricular arrhythmia in this patient population.

### 2.4. Statistical Analysis

Normality was tested with the Shapiro–Wilk test. Parameters are indicated as mean ± standard deviation (SD), median and ranges or as number and percentage, where appropriate. Between-group comparisons of continuous variables were performed by Student’s t-test, the factorial analysis of variance (ANOVA). Categorical variables were compared by Chi-square test. The *p*-value for statistical significance was set to *p* < 0.05.

The software SPSS (version 26, IBM (TM), Chicago, IL, USA) and JASP version 0.95.4 were used.

AI tools (Microsoft M365 Copilot and Perplexity AI (2026)) were used for language editing purposes only.

## 3. Results

### 3.1. Study Population

A total of 209 patients met the study’s inclusion criteria, of whom 80 were surgically treated patients with SVD. The anatomical features of this cohort are detailed in Table 1. For comparative analysis, 57 patients with secundum ASD treated surgically and 72 treated percutaneously were included. Baseline characteristics of all three cohorts at the time of intervention are detailed in Table 2. Surgical repair of SVD occurred from 1961 to 2017; secundum ASD closures: surgical 1959–2017 and percutaneous 1997–2018.

SVD patients undergoing surgery were significantly older than those with surgically treated secundum ASD (median age 28.5 vs. 13.5 years, Table 2, *p* < 0.001), while their age distribution resembled that of patients who underwent percutaneous ASD closure (median age 25 years, *p* = 0.955). Notably, a majority (70%) of SVD patients underwent surgery in adulthood, reflecting later clinical presentation. Within this cohort, 63% had surgery before age 40, while 37% were operated on at age 40 or older.

### 3.2. Latest Follow-Up

At latest follow-up, patient ages were comparable between groups (Table 3). However, the postoperative follow-up was significantly shorter among patients with SVD compared to the other groups as detailed in Table 3 (*p* < 0.001). At latest follow-up, most patients remained asymptomatic across all groups (Table 3). No significant differences were observed between groups regarding medication use, particularly the requirement for oral anticoagulation as details in Table 3 (*p* = 0.703).

The transthoracic echocardiography showed a significantly lower RV systolic function in SVD patients (Table 3). The presence of valvular disease and pulmonary hypertension were similar between groups (Table 3).

### 3.3. Late Postoperative Complications

Reoperation was required significantly more often in SVD patients (14%) than in those with surgically treated (4%) or percutaneously treated secundum ASD (4%) (*p* = 0.033), despite shorter follow-up period in the SVD cohort.

In the SVD cohort, reoperations occurred mainly in patients having surgery during childhood (*n* = 6/11 patients) at a median of 28 years (range 1–50 years) after index surgery. Specific indications for reoperation included superior vena cava obstruction in three patients, pulmonary vein obstruction in three patients, residual shunt due to missed anomalous pulmonary venous connection during the index surgery in four patients, and one case of cerclage removal. Superior vena cava and pulmonary venous obstruction occurred exclusively in the SVD group.

In the surgically treated secundum ASD, two patients required reintervention for residual shunt; one treated with surgery and one with percutaneous treatment. This required reintervention, which occurred at a mean age of 33.5 years in this cohort.

In the percutaneously treated secundum ASD group, two patients were treated for residual shunt (one with surgery and one with percutaneous device implantation) 2 years after the index procedure, and one required surgery due to device embolization one month after the index procedure.

### 3.4. Late Arrhythmia

At the latest follow-up the prevalence of arrhythmias did not differ between groups, despite marked differences in age (Table 4). However, the SVD group, as well as the surgically treated ASD, showed a higher prevalence of atrial flutter and atrial tachycardia than the percutaneously treated ASD cohort. The need for electrophysiological ablation procedures among SVD patients was significantly higher compared to those treated percutaneously for secundum ASD (*p* = 0.013); whereas, no significant difference was noted compared to the surgically treated ASD group (*p* = 0.405). The need for pacemaker implantation did not differ between groups even though the tendency was higher in the surgically treated groups, as shown in Table 4. The median age of pacemaker implantation was 51 years (range 7–63 years) in the SVD cohort, 30 years (range 7–52 years) in the surgically treated ASD group and 32 years (range 9–56 years) in the percutaneously treated ASD group.

## 4. Discussion

The present study provides two important messages:

(1) Surgically treated SVD patients have a higher reoperation rate than surgically or percutaneously treated ASD patients, reflecting the complexity of surgery due to heterogeneous anatomical presentations.
(2) Patients with surgically treated SVD and surgically treated secundum ASD show a high prevalence of reentry supraventricular arrhythmias and require more electrophysiological ablation procedures during follow-up compared with patients who underwent percutaneous ASD closure.

Patients with SVD associated with PAPVC present unique features and anatomical challenges which make this population at risk for early and late surgical complications. Furthermore, our study illustrated that SVD is typically detected at a significantly older age than secundum ASD. These findings underscore the inherent diagnostic complexities associated with SVD, highlighting the limitations of echocardiography as a sole imaging modality. Comprehensive multimodality imaging, particularly cardiac computed tomography, cardiac magnetic resonance imaging and right heart catheterization, is essential for achieving accurate diagnosis and optimal surgical planning. Vogel et al. systematically performed RHC in patients with SVD and compared them to secundum ASD patients. Their study demonstrated that individuals with SVD develop higher pulmonary artery pressures and vascular resistance at a younger age than those with secundum ASD, highlighting the need for distinct management strategies for this patient population [7]. The indication for surgery is primarily based on clinical and hemodynamic criteria, including the presence of a significant left-to-right shunt, typically with a Qp:Qs ratio > 1.5, and evidence of right heart cavity dilatation [5]. Surgery in this patient population has demonstrated excellent safety, with low rates of mortality (<1%) and morbidity [4,6].

In the present study, reoperation emerged as a significant long-term burden among patients with SVD, affecting 14% of the cohort, a rate that was markedly higher than that observed in patients with secundum ASD. This finding highlights the complexity of the surgical management required for SVD reflecting the challenges posed by heterogeneous anatomical presentations. Banka et al. similarly demonstrated that patients with inferior SVD experienced a higher incidence of residual significant shunt following surgery (requiring 11% reoperation in inferior SVD cases) compared to those with secundum ASD, a complication that could potentially be mitigated through meticulous preprocedural imaging [15]. Furthermore, individuals with inferior SVD had less favorable technical outcomes, including prolonged cardiopulmonary bypass and cross-clamp times, extended intensive care unit stays, and increased overall hospital length of stay relative to patients undergoing surgery for secundum ASD [15]. However, with ongoing advancements in surgical techniques and imaging modalities, the incidence of reoperation due to residual significant shunt or misdiagnosis is expected to decrease substantially.

The present study does not determine which surgical technique is most advantageous for patients with SVD. However, existing literature suggests that the Warden technique may be associated with lower rates of superior vena cava and pulmonary vein obstruction, as well as reduced incidence of sinus node dysfunction [16].

In the present study population, pulmonary vein obstruction and superior vena cava obstruction were each observed in 4% of SVD patients, which required reoperation. These rates are notably higher than those reported in previous studies [4,6]. Attenhofer Jost et al. reported no pulmonary vein nor superior vena cava obstruction [4]. A recent metanalysis on the long-term postoperative outcomes of SVD reported an incidence of 1.4% pulmonary vein obstruction and 1.76% superior vena cava obstruction [4]. Our findings could be explained by the longer follow-up period relative to previous research. Notably, reoperation occurred at a median of 28 years after index surgery. This emphasized the importance of long-term follow-up in this patient population in a specialized tertiary center.

The main burden associated with both SVD and secundum ASD is supraventricular arrhythmia, a topic extensively studied in the literature for secundum ASD population [17,18,19,20]. In ASD patients, arrhythmias are generally considered an acquired condition resulting from long-term right ventricular overload and remodeling, chamber enlargement, and surgical scarring [17]. In the present study, the SVD cohort exhibited a high rate of supraventricular arrhythmia, similar to that observed in the secundum ASD groups. These arrhythmias are likely a consequence of both the underlying pathology with inherent right cavities remodeling and surgical scarring of the right atrium. Previous studies report right heart chamber enlargement, elevated pulmonary artery pressure, and valvular disease, were identified as key factors associated with atrial fibrillation [17,21]. This highlights the benefit of early closure of the atrial defect before significant RV remodeling occurs. Given the time-dependent nature of atrial fibrillation and the shorter postoperative follow-up in the SVD cohort compared with the other groups, the observed prevalence may underestimate the long-term burden and could increase with longer post-operative follow-up.

Prior studies in patients with SVD and secundum ASD have reported a higher risk of atrial fibrillation among those undergoing surgery after the age of 40 [20]. The absence of this association in our cohort is likely attributable to the relatively young age at surgery, with a median operative age of 28 years. Gatzoulis et al. reported that late surgical intervention for secundum ASD does not influence the occurrence of late atrial fibrillation [17].

Pacemaker implantation was slightly higher in patients with SVD. Based on the surgical complexity and proximity to the atrioventricular conduction system, the prevalence of pacemaker implantation was expected to be higher in the SVD cohort than in the ASD cohort. The rates of pacemaker implantation in the surgically treated ASD and SVD are comparable to a previous large study [22]. The prevalence of pacemaker implantation previously reported in the percutaneously treated ASD ranges from 1 to 3%, which also corresponds to our findings [23]. Our findings may suggest that the need for pacemaker implantation may not only be related to the surgery itself but also to pathological changes affecting the conduction system in SVD patients due to right atrial and ventricular remodeling. To support this hypothesis, the median age for pacemaker implantation in the SVD cohort was 51 years (range 7 to 63 years).

Transcatheter devices, first introduced in 2014, have proven a favorable safety profile and represent a promising treatment option for selected SVD patients. However, comparative data on long-term outcomes between transcatheter therapy and the current gold standard treatment, meaning surgery, is not yet available. Future and ongoing prospective studies comparing percutaneous SVD closure to surgical SVD closure are valuable to further define the role of this technique in clinical practice [24]. The possible complications related to percutaneous closure of SVD currently reported are device embolization, residual shunt and thrombosis [25]. The arrhythmic consequences of deploying transcatheter devices in the right atrium are not yet known.

The findings of our study enhance the current understanding of SVD patients, by providing additional insights into their characteristics and long-term surgical outcomes.

### Limitations

The major limitation, due to the rarity of this congenital anomaly, is the retrospective design of our study. Each center had its own individualized care management for SVD patients. The latter leads to a lack of uniformity in our database. Another limitation is the shorter postoperative follow-up period observed in patients with SVD compared to the other groups, despite similar ages across cohorts at latest follow-up. Given that arrhythmic and reoperation events are time-dependent, a time-to-event analysis would have been preferable. Nevertheless, despite the shorter available postoperative follow-up in the SVD cohort, the crude reoperation rate was higher in patients with SVD than in patients with surgically or percutaneously treated secundum ASD. This finding supports the complexity of accurate diagnosis, surgical planning, and surgical repair in this population; although, the absence of time-to-event data limits interpretation of the comparative long-term risk.

As some patients in the surgically treated SVD and ASD groups underwent surgery as early as the 1950s and 1960s. Over this period, both surgical techniques and preoperative imaging modalities have evolved substantially. Therefore, outcomes observed in patients treated in earlier eras may not fully reflect those expected with contemporary diagnostic and surgical approaches.

The arrhythmic comparison between groups was limited by the small number of events, heterogeneous rhythm surveillance, and unequal follow-up duration; therefore, the absence of statistically significant differences should not be interpreted as evidence of equivalent arrhythmic risk. In addition, electrophysiological studies and ablation procedures may reflect clinical referral patterns, center-specific practice, and differences in surveillance intensity, and therefore should not be interpreted as direct surrogates of arrhythmic burden.

These limitations illustrate the complexity and heterogeneity of SVD and the need for evidenced care management in these patients. Despite these challenges, the present study provides clinical data on a decent number of patients with SVDs.

## 5. Conclusions

Patients with a surgically treated SVD had a higher reoperation rate compared to those treated for secundum ASD, reflecting the greater surgical complexity. Moreover, patients with surgically treated SVD exhibited a substantial burden of reentry arrhythmias, and a high rate of electrophysiological studies. Given these findings, long-term follow-up is essential to monitor for reintervention and arrhythmic complications in this patient population.

## Figures and Tables

**Table 1:** Anatomical features of the SVD and surgical technique.

	Characteristics of Patients with Sinus Venosus Defect, *n* = 80
Number of anomalous pulmonary venous connection	
1, *n* (%)	29 (36)
2, *n* (%)	42 (52)
3, *n* (%)	4 (5)
Which pulmonary veins anomalously connected	
RUPV	69 (86)
RMPV	14 (18)
RLPV	9 (11)
LUPV	6 (8)
LLPV	2 (3)
Persistent left superior vena cava, *n* (%)	16 (20)
Surgical technique	
Single-patch	16 (20)
Double-patch	40 (50)
Warden	16 (20)
Unknown	8 (10)

SVD: sinus venosus defect; RUPV: right upper pulmonary vein; RMPV: right middle pulmonary vein; RLPV: right left pulmonary vein; LUPV: left upper pulmonary vein; LLPV: left lower pulmonary vein.

**Table 2:** Characteristics at index SVD and ASD closure.

	Surgically Treated SVD, *n* = 80	Surgically Treated ASD, *n* = 57	Percutaneously Treated ASD, *n* = 72	*p*-Value
Qp:Qs ratio at correction measured on RHC and/or TTE	2.3 (1.0–7.0)	2.1 (1.4–4.5)	1.6 (0.9–2.8)	<0.001
Dilated right ventricle, *n* (%)	56 (70)	20 (35)	40 (56)	0.001
Preoperative pulmonary hypertension measured on TTE	11 (14)	6 (11)	7 (10)	0.713
Age at surgical or percutaneous treatment, y	28.5 (2–68)	13.5 (0–48)	25 (3–75)	<0.001
Concomitant tricuspid annuloplasty during index surgery, *n* (%)	4 (5)	2 (4)	-	
Concomitant pulmonary valve commissurotomy during index surgery, *n* (%)	1 (1)	0	-	
Concomitant biatrial maze treatment during index surgery	1 (1)	-	-	
Concomitant cryotherapy of the cavo tricuspid isthmus	1 (1)	-	-	

Data are mean ± standard deviation, median and range, when appropriate or *n* (%). SVD: sinus venosus defect; ASD: atrial septal defect.

**Table 3:** Clinical data during latest follow-up.

	Surgically Treated SVD, *n* = 80	Surgically Treated ASD, *n* = 57	Percutaneously Treated ASD, *n* = 72	*p*-Value
Women, *n* (%)	44 (55)	30 (52)	46 (64)	0.376
Age at latest follow-up, y	38.5 (18–75)	29.5 (18–88)	30 (18–82)	0.103 *
Time since surgery, y	2.5 (1 month–55 years)	18 (1 month–55 years)	5 (1 month–55 years)	<0.001 †
Symptoms of latest follow-up	26 (33)	20 (35)	23 (32)	0.924
- Dyspnoea	9 (11)	5 (8)	9 (13)	0.714
- Palpitations	17 (21)	11(19)	10 (14)	0.485
- Chest pain	4 (5)	4 (7)	6 (8)	0.709
Treatment, *n* (%)	38 (48)	17 (30)	26 (36)	0.095
- ACE Inhibitor, *n* (%)	4 (5)	2 (3)	3 (4)	0.912
- Angiotensin II receptor blocker, *n* (%)	6 (8)	3 (5)	3 (4)	0.667
- Anticoagulation, *n* (%)	16 (20)	9 (15)	11 (15)	0.703
- Antiplatelets, *n* (%)	17 (21)	5 (9)	10 (14)	0.124
- Betablockers, *n* (%)	19 (23)	5 (9)	12 (17)	0.072
Echocardiography at latest follow-up	(*n* = 79, 99%)	(*n* = 55, 96%)	(*n* = 71, 99%)	
Right atrium dilatation, *n* (%) Missing data (*n*)	27 (33) 13	12 (21) 9	16 (22) 10	0.117
Right ventricle dilatation, *n* (%) Missing data (*n*)	29 (36) 0	16 (28) 1	17 (23) 1	0.248
Right ventricular systolic function				
- S’ wave, cm/s - TAPSE, mm	9.8 ± 2.5 16.4 ± 3.7	10.5 ± 2.1 18.1 ± 3.9	11.9 ± 2.6 22 ± 5.9	<0.001 § <0.001 ß
Missing data (*n*)	28	18	28	
Systolic pulmonary artery pressures, mmHg Missing data (*n*)	25 (16–47) 27	23 (11–50) 24	25 (8–49) 36	0.740
Pulmonary hypertension based on echocardiography	3 (3)	2 (4)	5 (7)	0.577
Valvular disease Missing data (*n*)	12 0	13 1	18 1	0.256

Data are mean ± standard deviation, median and range, when appropriate or *n* (%). SVD: sinus venosus defect; ASD: atrial septal defect; TAPSE: Tricuspid Annular Plane Systolic Excursion. * surgically treated SVD vs. surgically treated ASD (*p* = 0.54); surgically treated SVD vs. percutaneously treated ASD (*p* = 0.089); surgically treated ASD vs. percutaneous treated ASD (*p* = 0.775). † surgically treated SVD vs. surgically treated ASD (*p* = 0.004); surgically treated SVD vs. percutaneously treated ASD (*p* = 0.011); surgically treated ASD vs. percutaneously treated ASD (*p ≤* 0.001). § surgically treated SVD vs. surgically treated ASD (*p* = 0.205); surgically treated SVD vs. percutaneously treated ASD (*p* < 0.001); surgically treated ASD vs. percutaneously treated ASD (*p ≤* 0.013). ß surgically treated SVD vs. surgically treated ASD (*p* = 0.042); surgically treated SVD vs. percutaneously treated ASD (*p* < 0.001); surgically treated ASD vs. percutaneously treated ASD (*p* ≤ 0.001).

**Table 4:** Prevalence of late arrythmia during follow-up.

	Surgically Treated SVD, *n* = 80	Surgically Treated ASD, *n* = 57	Percutaneously Treated ASD, *n* = 72	*p*-Value
Atrial fibrillation, *n* (%)	7 (9)	8 (14)	7 (10)	0.588
Atrial flutter, *n* (%)	7 (9)	3 (5)	1 (1)	0.128
Atrial tachycardia, *n* (%)	11 (14)	6 (11)	5 (7)	0.394
Electrophysiological study, *n* (%)	9 (11)	4 (7)	1 (1)	0.052 *
Pacemaker implantation, *n* (%)	7 (9)	4 (7)	2 (3)	0.301 †

SVD: sinus venosus defect; ASD: atrial septal defect. * Electrophysiological study: surgically treated SVD vs. surgically treated secundum ASD *p* = 0.405; surgically treated SVD vs. percutaneously treated secundum ASD *p* = 0.013; surgically treated ASD vs. percutaneously treated ASD (*p* = 0.093). † Need for pacemaker implantation: surgically treated SVD vs. surgically treated secundum ASD *p* = 0.713; surgically treated SVD vs. percutaneously treated secundum ASD *p* = 0.110; surgically treated ASD vs. percutaneously treated ASD (*p* = 0.242).

## Data Availability

The data supporting the findings of this study are available from the corresponding author upon reasonable request. The data are not publicly available because of privacy and ethical restrictions.
